# *Her2*^Ile^655^Val^ polymorphism and its association with breast cancer risk: an updated meta-analysis of case-control studies

**DOI:** 10.1038/s41598-018-25769-y

**Published:** 2018-05-09

**Authors:** B. Madhu Krishna, Sanjib Chaudhary, Aditya K. Panda, Dipti Ranjan Mishra, Sandip K. Mishra

**Affiliations:** 1 Cancer Biology Lab, Gene Function & Regulation Group, , Institute of Life Sciences, Nalco square, Chandrasekharpur, Bhubaneswar, 751023 Odisha India; 2 Centre for Life Sciences, Central University of Jharkhand, Ranchi, 835205 Jharkhand India; 3 Gene Function & Regulation Group, Institute of Life Sciences, Nalco square, Chandrasekharpur, Bhubaneswar, 751023 Odisha India

## Abstract

Breast cancer (BC) is one of the most common types of cancer in women worldwide. Several factors including genetic and environmental have been linked with susceptibility to development of BC. *Her2* is a transmembrane protein with tyrosine kinase activity, overexpressed in several cancers including BC. Various studies in different populations have shown association of *Her2* variants with susceptibility to BC, however these results were inconsistent, inconclusive and controversial. To obtain a common conclusive finding, we performed meta-analysis of 35 case-control studies reported earlier including 19, 220 cases and 22, 306 controls. We observed significant association of *Her2* ^Ile^655^Val^ polymorphism with susceptibility to development of breast cancer (Overall allele Val vs Ile: OR = 1.130, 95% CI = 1.051–1.216, p = 0.001; Ile-Val vs Ile-Ile: OR = 1.100, 95% CI = 1.016–1.192, p = 0.019; Val-Val+Ile-Val vs Ile-Ile: OR = 1.127, 95% CI = 1.038–1.223, p = 0.004). Subgroup analysis indicated a significant association with susceptibility to breast cancer in African and Asian populations. However, such association was not observed in other ethnic groups. Our findings suggested that *Her2* ^Ile^655^Val^ polymorphism is associated with breast cancer risk in overall, Asian and African populations, and can be used as diagnostic marker for BC.

## Introduction

Breast cancer (BC) is second leading cause of cancer deaths worldwide and approximately 1.7 million new cases are being diagnosed every year and 521,900 deaths occurred in 2012 alone globally^1^. It has been estimated that 252,710 new cases of invasive breast cancer will be diagnosed in 2017 among women in US alone. Although, breast cancer is most common in females it also rarely diagnosed in male individuals and 2,470 males are estimated to be diagnosed with breast cancer in 2017 in United States^2^. Among the overall cancer deaths worldwide, approximately 60% of deaths occur in developing countries including India. In Indian woman, majority of cancer related deaths are due to breast cancer^1^.

BC is highly heterogeneous and ~60–70% is of estrogen receptor positive which responds to anti-hormone therapy^3^. Estrogen receptor (ER) plays an important role in breast cancer progression and treatment. Approximately 20–30% breast cancers are of Human epidermal growth factor receptor2 (*Her2*) positive and are highly aggressive in nature^4^. High levels of *Her2* expression was also observed in tamoxifen resistant breast cancers. Human epidermal growth factor receptor family members are a group of molecules having tyrosine kinase activity with no natural ligand found till date. Heterodimerization among the family members leads to autophosphorylation of cytoplasmic domain which leads to cell proliferation^5–7^. *Her2* is highly expressed in various cancers types viz. breast, endometrial, ovarian, colon, lung, prostrate and cervical cancers. Role of ERBB2/Her2 in physiological processes (cell growth, differentiation and tissue development) as well as in carcinogenesis and metastasis has been well investigated^8–10^. Her2 plays major role in the regulation of several pathways such as Raf/Ras/MAPK and PI3K/AKT pathways^11^. Receptor mediated signaling pathways has pivotal role in the regulation of normal cell function, growth and division. However disruption of these pathways might lead to several cancers^12–15^. Her2 positive breast cancers show poor survival rate, treatment with tyrosine inhibitors showing promising results in harboring these aggressive tumors^16^. Trastuzumab a monoclonal antibody specifically binds to Her2 and disrupts the downstream pathways of Her2 and it is effectively used for the treatment of Her2 positive breast cancers^17–19^. However several patients developed resistance to trastuzumab over a period of time^20^. Recent studies suggested that Her2 ^Ile^655^Val^ polymorphism is associated with cardiac toxicity. Moreover, it has been identified that both the Her2 ^Ala^1170^Pro^ polymorphisms also responsible for increasing the risk of cardiac toxicity in women administrated with trastuzumab^21,22^.

Genetic epidemiological studies indicated association between single nucleotide polymorphisms and different cancers^23–25^. Cell cycle regulatory role of Her2 and its importance in prognosis of breast cancer clearly indicates that polymorphism in coding region of *Her2* might be associated with either cancer susceptibility risk or resistance. One such single nucleotide transition mutation in transmembrane domain coding region of *Her2* at codon 655 [Isoleucine (Ile) to Valine (Val) mutation, *Her2* ^Ile^655^Val^] was well investigated in different populations in relation to risk of breast cancer^26–61^. Milikan *et al*.^40^ reported the association of Valine allele at *Her2* 655 codon with breast cancer risk. Whereas, Baxter *et al*.^29^ and Xie *et al*.^60^ found no association with breast cancer risk in women aged <40 years, post menopausal respectively. However, few researchers performed meta-analysis and tried to conclude the possible correlation of *Her2* polymorphism with breast cancer risk. Tao *et al*.^56^ showed no association in overall analysis, however mild association of *Her2* polymorphism with susceptibility to breast cancer in Asian ethnic group was suggested. Another meta-analysis by Chen *et al*.^62^ including 32 case control studies revealed comparable distribution of *Her2* ^Ile^655^Val^ variants among cases and controls in Caucasian, American and European population. Interestingly, Asian ethnic group showed significant association of breast cancer risk with *Her2* ^Ile^655^Val^ polymorphism. In the present meta-analysis, total of 35 case-control studies were analyzed and investigated for possible association of *Her2* ^Ile^655^Val^ polymorphism with development of breast cancer. Furthermore, we subgrouped included reports according to ethnicity and the association was analyzed .

## Results

### Characteristics of eligible studies

To understand association of *Her2* ^Ile^655^Val^ polymorphism with breast cancer risk, we have performed meta-analysis using 35 case-control eligible studies including 19, 220 cases and 22, 306 controls. Genotype and allele frequency for case and control of each eligible study was extracted and the characteristics of each study are shown in Table 1. For subgroup analysis the identified studies were categorized based on their ethnicity viz. Caucasian, American, Afro-American, African, European and Asian respectively.

**Table 1:** Characteristics and distribution of Her2 polymorphism in each study involved in meta-analysis.

**S.No**	**First Author**	**Year**	**Ethnic group**	**Cancer type**	**Case**	**Control**	**HWE**	**Genotype Distribution**				**Allele Distribution (%)**				**Genotyping method**
								**Case**		**Control**		**Case**		**Control**		
								**Ile/Ile**	**Ile/Val + Val/Val**	**Ile/Ile**	**Ile/Val + Val/Val**	**Ile**	**val**	**Ile**	**Val**	
1	AbdRaboh NR *et al*.	2013	Egyptian	BC	64	86	Y	39	25	67	19	99	29	152	20	PCR-RFLP
2	Al-Janabi AM *et al*.	2015	Iraqi	BC	300	200	Y	141	159	120	80	407	193	308	92	PCR-RFLP
3	Akisik E *et al*.	2004	Turkish	BC	121	145	Y	98	23	117	28	218	24	260	30	PCR-RFLP
4	An HJ *et al*.	2005	Korean	BC	177	126	Y	139	38	96	30	311	43	221	31	PCR-RFLP
5	Baxter SW *et al*.	2001	Caucasian	BC	315	256	Y	190	125	138	118	489	141	377	135	PCR-RFLP
6	Benusiglio PR *et al*.	2006	British	BC	1989	2155	Y	1128	861	1230	925	3004	974	3251	1059	Taqman
7	Carrillo-Moreno DI *et al*.	2016	Mexican	BC	400	225	Y	312	88	191	34	709	91	415	35	Taqman
8	Cox DG *et al*.	2005	Cohort	BC	1313	1717	Y	766	505	980	687	1979	563	2551	783	Taqman
9	Frank B *et al*.	2005	German	BC	347	960	Y	186	161	525	435	504	190	1427	493	Taqman
10	GENICA *et al*.	2010	Caucasian	BC	3138	5486	Y	1856	1282	3072	2414	4795	1481	8227	2745	MALDI‐TOF MSa and PCR‐based fragment analyses
11	Hishida A *et al*.	2002	Japanese	BC	236	184	Y	182	54	136	48	415	57	313	55	Not reported
12	Kalemi TG *et al*.	2005	Greek	BC	42	51	N	32	10	36	15	74	10	87	15	PCR-RFLP
13	Kallel I *et al*.	2010	Tunician	BC	148	290	N	130	18	240	50	274	22	530	50	PCR-RFLP
14	Kamali-Sarvestani E *et al*.	2004	Iranian	BC	204	138	Y	145	59	102	36	347	61	236	40	PCR-RFLP
15	Kara N *et al*.	2010	Turkish	BC	204	192	Y	153	51	141	51	352	56	330	54	PCR-RFLP
16	Keshava C *et al*. (a)	2001	Caucasian	BC	89	180	Y	59	30	129	51	144	34	302	58	PCR-RFLP
17	Keshava C *et al*. (b)	2001	African- American	BC	34	63	Y	32	2	57	6	66	2	120	6	PCR-RFLP
18	Keshava C *et al*. (c)	2001	Latinos	BC	28	77	Y	17	11	58	19	44	12	134	20	PCR-RFLP
19	Lee SC *et al*.	2008	Taiwan	BC	424	318	Y	341	83	273	45	762	86	590	46	PCR-RFLP
20	Millikan R *et al*. (a)	2003	African- American	BC	754	676	N	658	96	606	70	1404	104	1282	70	Taqman
21	Millikan R *et al*. (b)	2003	Whites	BC	1261	1132	N	752	509	684	448	1933	589	1743	521	Taqman
22	Montgomery KG *et al*.	2003	Australian	BC	409	299	Y	240	169	196	103	618	200	486	112	Dual color allele‐specific PCR assay
23	Mutluhan H *et al*.	2008	Turkish	BC	166	208	Y	128	38	166	42	290	42	372	44	PCR-RFLP
24	Naidu R *et al*.	2008	Malaysian	BC	230	200	Y	165	65	159	41	387	73	355	45	PCR-RFLP
25	Nelson SE *et al*.	2005	Europian	BC	1094	976	Y	637	457	551	425	1670	518	1458	494	Taqman
26	Ozturk O *et al*.	2013	Turkish	BC	118	118	N	61	57	87	41	179	57	215	41	PCR-RFLP
27	Papadopoulou E *et al*.	2007	Greek	BC	56	45	Y	15	41	19	26	52	60	54	36	PCR-RFLP
28	Parvin S *et al*.	2016	Asian	BC	310	250	Y	210	100	189	61	508	112	433	67	PCR-RFLP
29	Pinto D *et al*.	2004	Portuguese	BC	152	146	Y	88	64	107	39	233	71	249	43	PCR-RFLP
30	Qu S *et al*.	2008	Chineese	BC	3012	3004	Y	2298	714	2252	752	5244	780	5191	817	Taqman
31	Rajkumar T *et al*.	2008	South Indian	BC	250	500	Y	181	69	363	137	424	76	845	155	Taqman
32	Sezgin E *et al*.	2011	Turkish	BC	58	55	Y	44	14	37	18	102	14	91	19	PCR-RFLP
33	Siddig A *et al*.	2008	Sudan	BC	68	81	Y	56	12	75	6	123	13	155	7	Taqman
34	Tommasi S *et al*.	2007	Caucasian	BC	628	169	Y	433	195	125	44	947	209	291	47	Taqman
35	Wang-Gohrke S *et al*.	2001	Caucasian	BC	615	1078	Y	360	255	646	432	939	291	1666	490	PCR-RFLP
36	Watrowski R *et al*.	2015	Austrian	BC	80	100	Y	51	29	63	37	128	32	160	40	Taqman
37	Xie D *et al*.	2000	Chineese	BC	339	359	Y	243	96	280	79	571	107	638	80	PCR-RFLP
38	ŽÚBOR P *et al*.	2006	Slovak republican	BC	47	60	Y	22	25	42	18	66	28	101	19	PCR-RFLP

Keshava *et al*. Caucasian ethnic group designated as (a), African-American ethnic group designated as (b) and Latinos ethnic group designated as (c). Millikan *et al*. African-American ethnic group designated as (a) and whites designated as (b).

### Heterogeneity test

To evaluate the heterogeneity among the studies Q test with I^2^ statistics were used. I^2^ more than 50 (I^2^ > 50) with significant p-value (p < 0.05) considered to be presence of heterogeneity among included studies. Among the models tested, heterogeneity was observed in allele comparison, heterozygous and dominant genetic models. However, other genetic comparison models such as recessive and homozygous were homogeneous. Observations of heterogeneity Q test and I^2^ statistics of each model are shown in Table 2. Based on results of heterogeneity test, fixed or random effect model was used for meta-analysis.

**Table 2:** Statistics for heterogeneity analysis and publication bias.

S.no	Model	Heterogeneity analysis			Egger’s regression			Publication bias	Fixed/Random
1	Overall allele Val vs. Ile	**Q-value**	**P heterogeneity**	**I** ^**2**^ **value**	**Intercept**	**95% CI**	**p-value**	Imputed	Random
		95.232	0.000	61.147	1.46746	0.764–2.170	0.00015		
2	Homozygous Val-Val vs. Ile-Ile	54.756	0.014	37.906	0.88689	0.318–1.455	0.00324	Imputed	Fixed
3	Heterozygous Ile-Val vs. Ile-Ile	76.010	0.000	51.322	1.26086	0.593–1.928	0.00049	Imputed	Random
4	Recessive Val-Val vs. Ile-Ile + Ile-Val	47.555	0.061	28.503	0.74160	0.197–1.285	0.00907	Imputed	Fixed
5	Dominant Val-Val + Ile-Val vs. Ile-Ile	87.290	0.000	57.612	1.42523	0.730–2.119	0.00019	Imputed	Random

### Publication bias

Begg’s funnel plot and egger’s regression test was performed to assess the publication bias within the studies included in meta-analysis. Results are imputed in Table 2. We observed significant publication bias in all genetic models tested and were resolved by “trim and fill” technique (Supplementary Fig. 1).

### Statistical analysis

**Figure 1:**
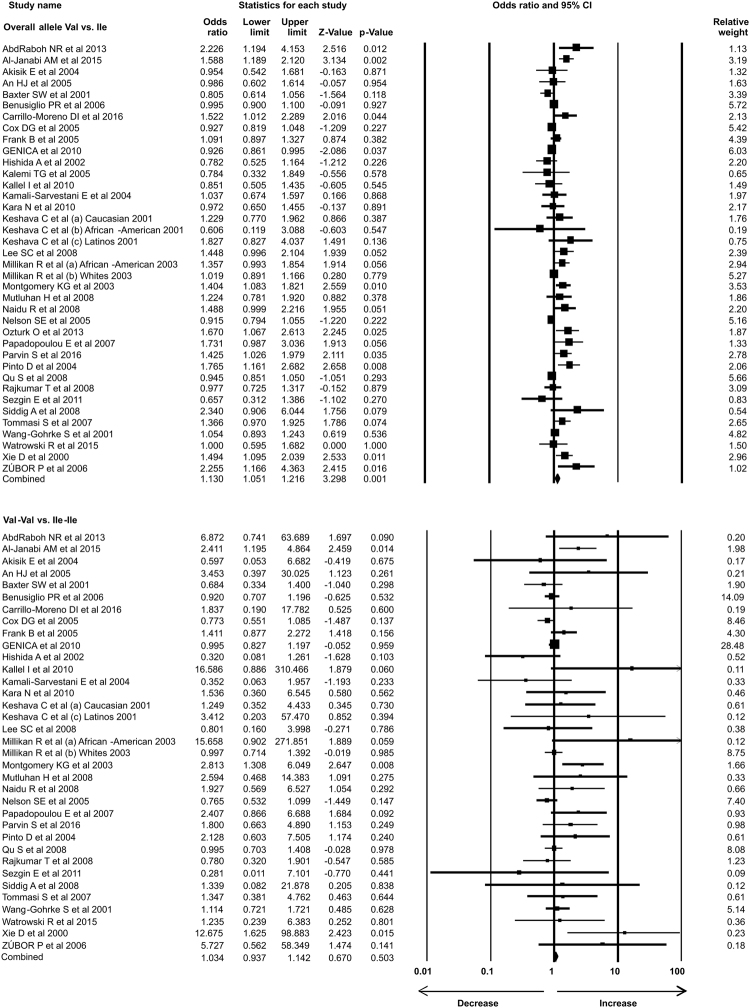
Forest plot: Overall allele and genotypic (Homozygous: Val-Val vs. Ile-Ile) analysis of *Her2* ^Ile^655^Val^ (rs1136201) gene polymorphism and validation of it’s association with breast cancer risk. Black squares represent the value of OR and horizontal line indicates 95% Confidence Interval (CI) of odds ratio (OR).

**Figure 2:**
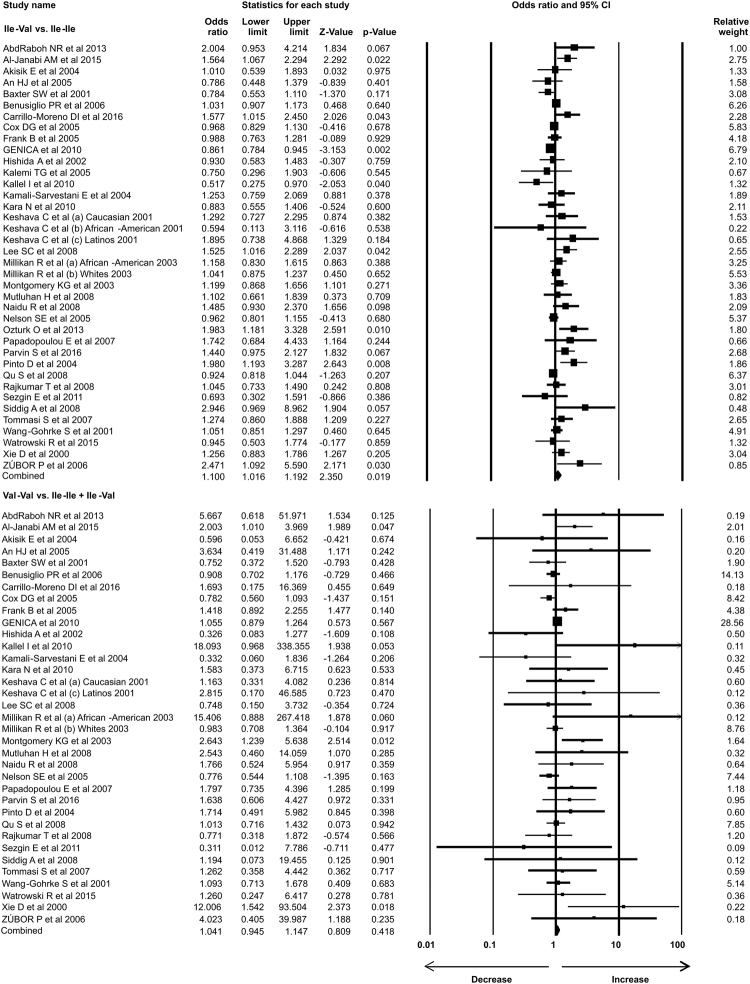
Forest plot: Genotypic (Heterozygous: Ile-Val vs Ile-Ile and Recessive: Val-Val vs. Ile-Ile + Ile-Val) analysis of *Her2* ^Ile^655^Val^ (rs1136201) gene polymorphism and investigation of it’s association with breast cancer risk using OR with 95% CI.

**Figure 3:**
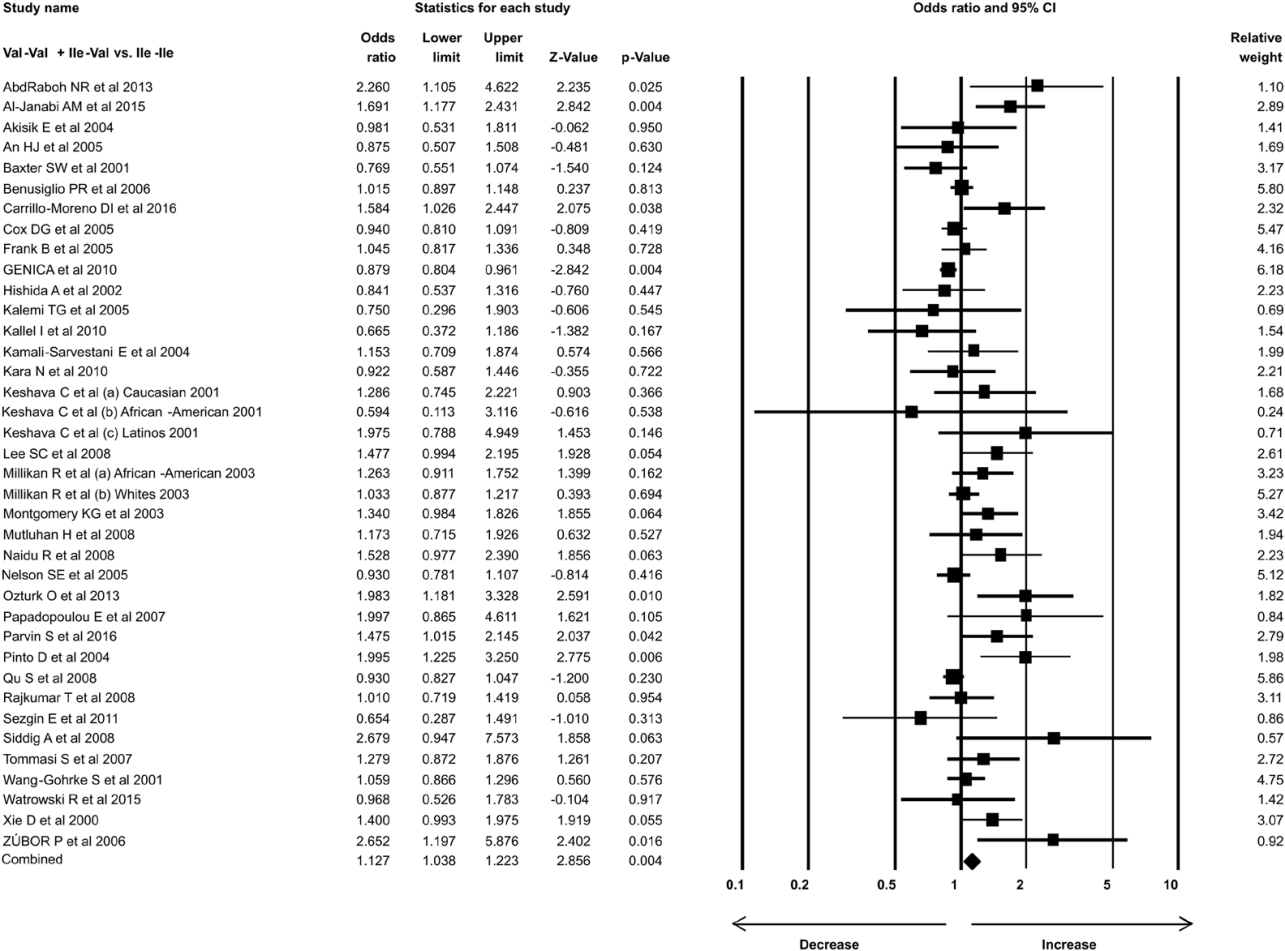
Forest plot: Genotypic (Dominant: Val-Val + Ile-Val vs Ile-Ile) analysis of *Her2* ^Ile^655^Val^ (rs1136201) gene polymorphism and evaluation of its association with increased risk of breast cancer. Black squares represent the value of OR and horizontal line indicates 95% Confidence Interval (CI) of odds ratio (OR).

**Figure 4:**
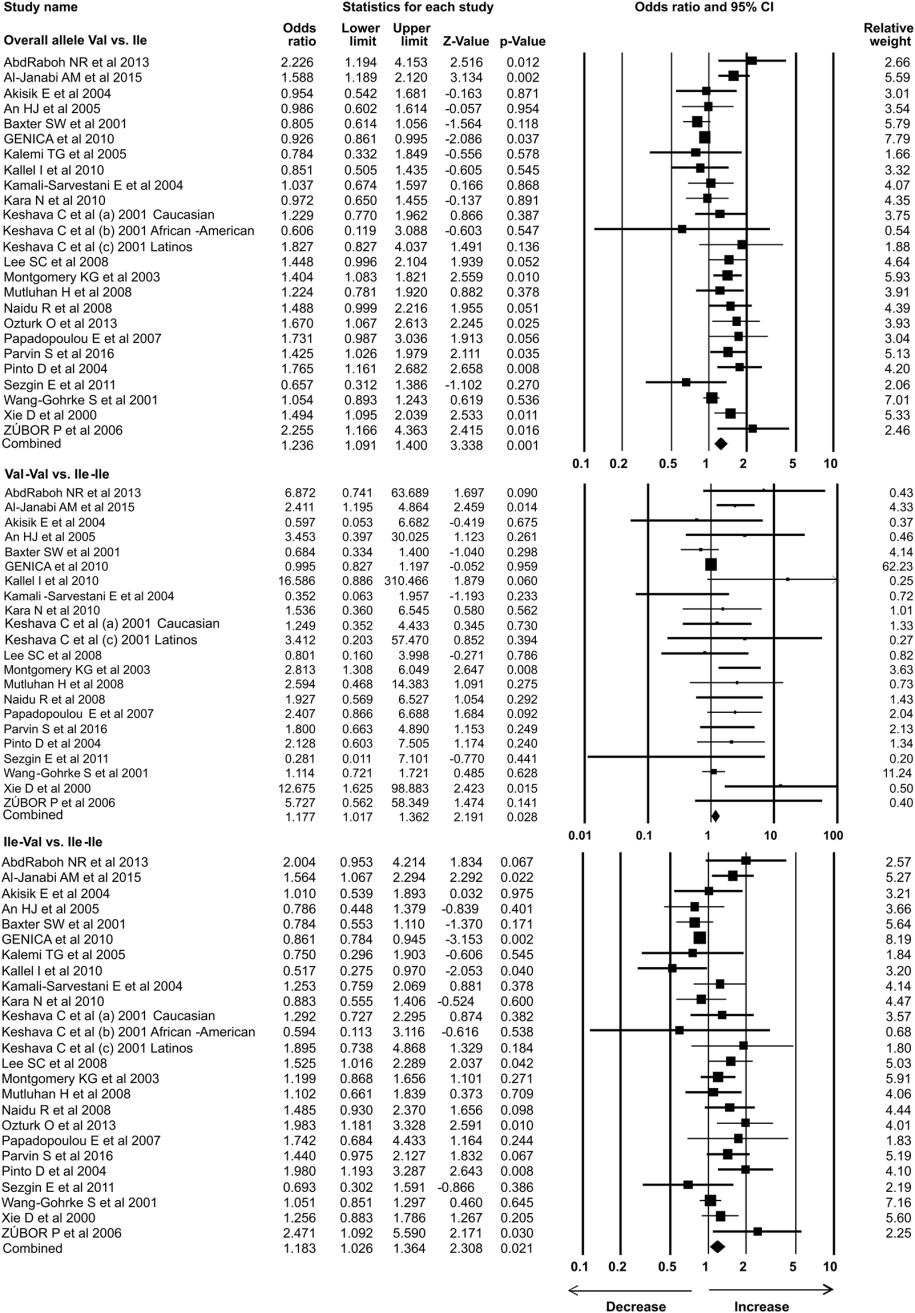
Forest plot: Overall allele and genotypic (Homozygous: Val-Val vs. Ile-Ile and Heterozygous: Ile-Val vs Ile-Ile) analysis of studies in which RFLP used as detection method for *Her2* ^Ile^655^Val^ (rs1136201) gene polymorphism and evaluation of its association with breast cancer risk. Black squares represent the value of OR and horizontal line indicates 95% Confidence Interval (CI) of odds ratio (OR).

**Figure 5:**
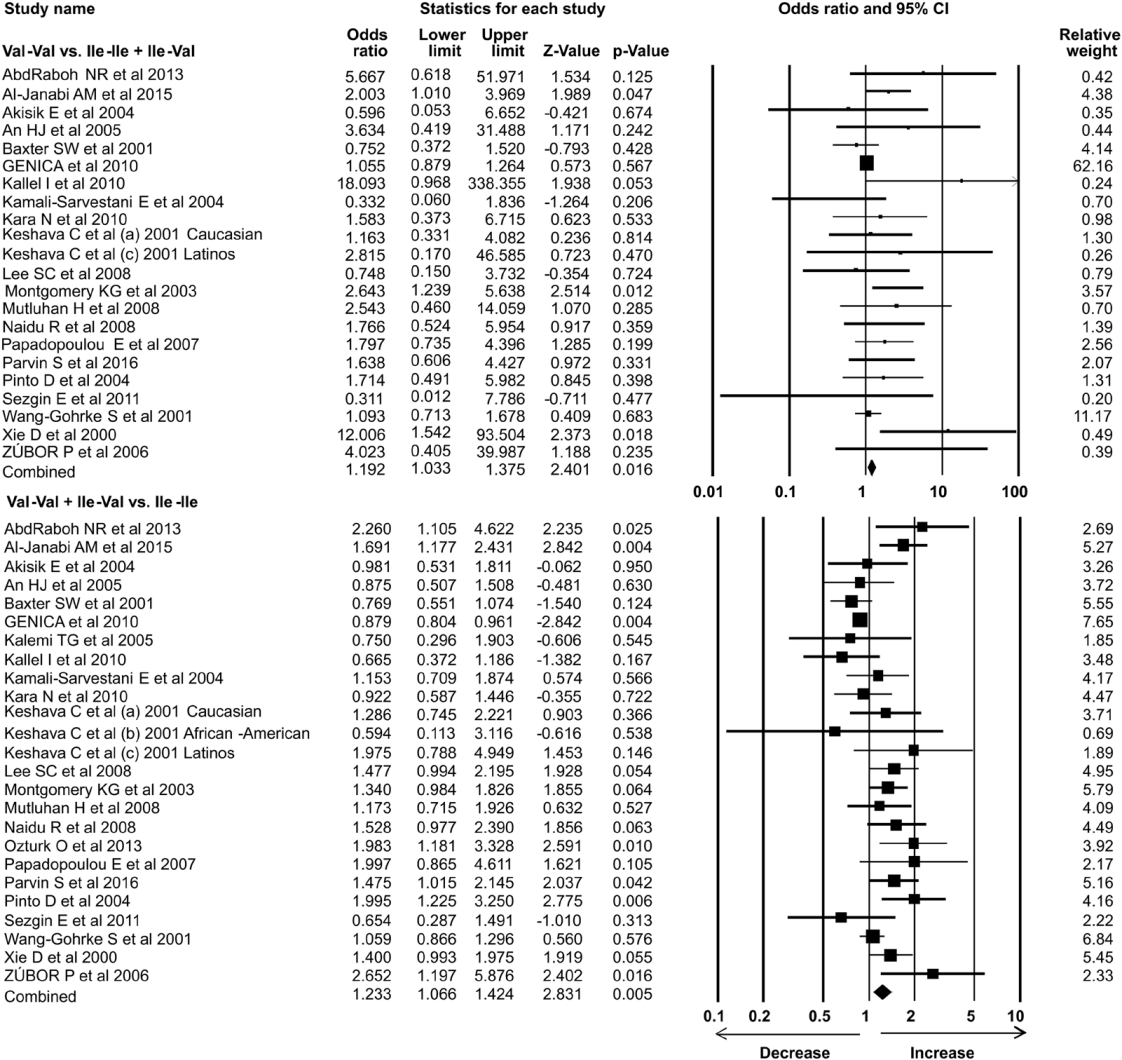
Forest plot: Genotypic (Recessive: Val-Val vs. Ile-Ile + Ile-Val; Dominant: Val-Val + Ile-Val vs Ile-Ile) analysis of studies in which RFLP used as detection method for *Her2* ^Ile^655^Val^ (rs1136201) gene polymorphism and evaluation of its association with increased risk of breast cancer. Black squares represent the value of OR and horizontal line indicates 95% Confidence Interval (CI) of odds ratio (OR).

**Figure 6:**
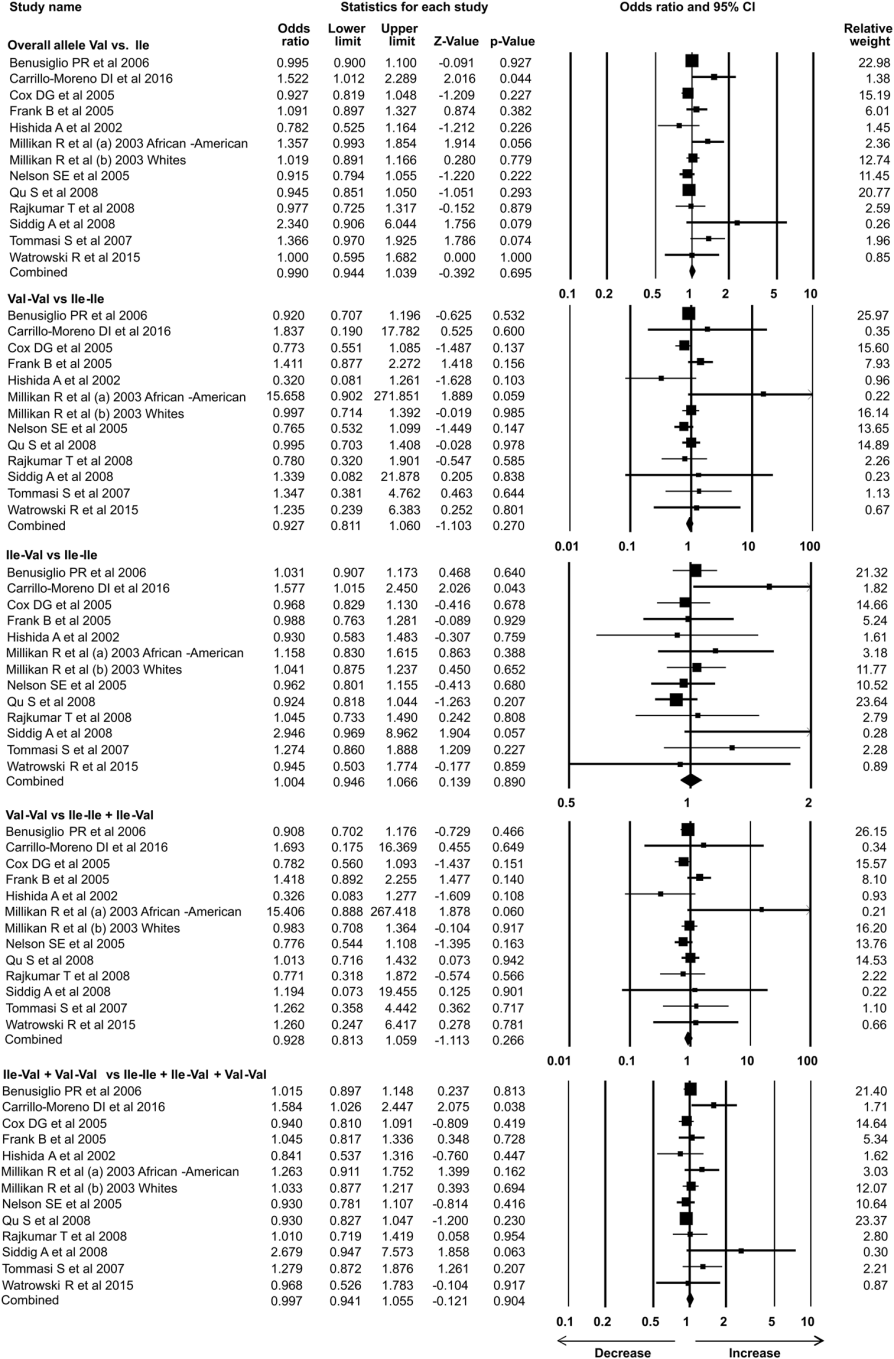
Forest plot: Overall allele and genotypic analysis of studies in which Taqman used as detection method for *Her2* ^Ile^655^Val^ (rs1136201) gene polymorphism and evaluation of its association with breast cancer risk. Black squares represent the value of OR and horizontal line indicates 95% Confidence Interval (CI) of odds ratio (OR). Overall allele Val vs Ile: OR = 0.990, 95% CI = 0.944–1.039, p = 0.695; Homozygous Val-Val vs Ile-Ile: OR = 0.927, 95% CI = 0.811–1.060, p = 0.270; Heterozygous Ile-Val vs Ile-Ile: OR = 1.004, 95% CI = 0.946–1.066, p = 0.890; Recessive Val-Val vs Ile-Ile + Ile-Val: OR = 0.928, 95% CI = 0.813–1.059, p = 0.266; Dominant model Val-Val + Ile-Val vs Ile-Ile: OR = 0.997, 95% CI = 0.941–1.055, p = 0.904.

In the present study 35 case-control studies were included and cumulative analysis demonstrated the association of *Her2* polymorphism with increased risk of breast cancer. The overall allele model revealed association between *Her2* polymorphism and breast cancer risk with Odds ratio (OR) = 1.130, 95% confidence interval (CI) = 1.051–1.216, p = 0.001. Furthermore, both dominant and heterozygous models showed significant association of Her2 ^Ile^655^Val^ polymorphism with increased risk of breast cancer (Dominant model Val-Val + Ile-Val vs Ile-Ile: OR = 1.127, 95% CI = 1.038–1.223, p = 0.004; Heterozygous Ile-Val vs Ile-Ile: OR = 1.100, 95% CI = 1.016–1.192, p = 0.019). However, comparison of genotypes in other genetics models didn’t show significant association (Homozygous Val-Val vs Ile-Ile: OR = 1.034, 95% CI = 0.937–1.142, p = 0.503; Recessive Val-Val vs Ile-Ile + Ile-Val: OR = 1.041, 95% CI = 0.945–1.147, p = 0.418) (Figs 1–3). Furthermore, Studies were grouped based on the techniques used for the detection of polymorphism and were analyzed for the association with breast cancer. Studies which used RFLP method as genotypic detection were showing significant association with breast cancer risk in all the models (Overall allele Val vs Ile: OR = 1.236, 95% CI = 1.091–1.400, p = 0.001; Homozygous Val-Val vs Ile-Ile: OR = 1.177, 95% CI = 0.017–1.362, p = 0.028; Heterozygous Ile-Val vs Ile-Ile: OR = 1.183, 95% CI = 1.026–1.364, p = 0.021; Recessive Val-Val vs Ile-Ile + Ile-Val: OR = 1.192, 95% CI = 1.033–1.375, p = 0.016; Dominant model Val-Val + Ile-Val vs Ile-Ile: OR = 1.233, 95% CI = 1.066–1.424, p = 0.005) (Figs 4 and 5). However, the studies in which Taqman used as detection method showed no association with increased risk of breast cancer (Fig. 6).

### Subgroup analysis

**Figure 7:**
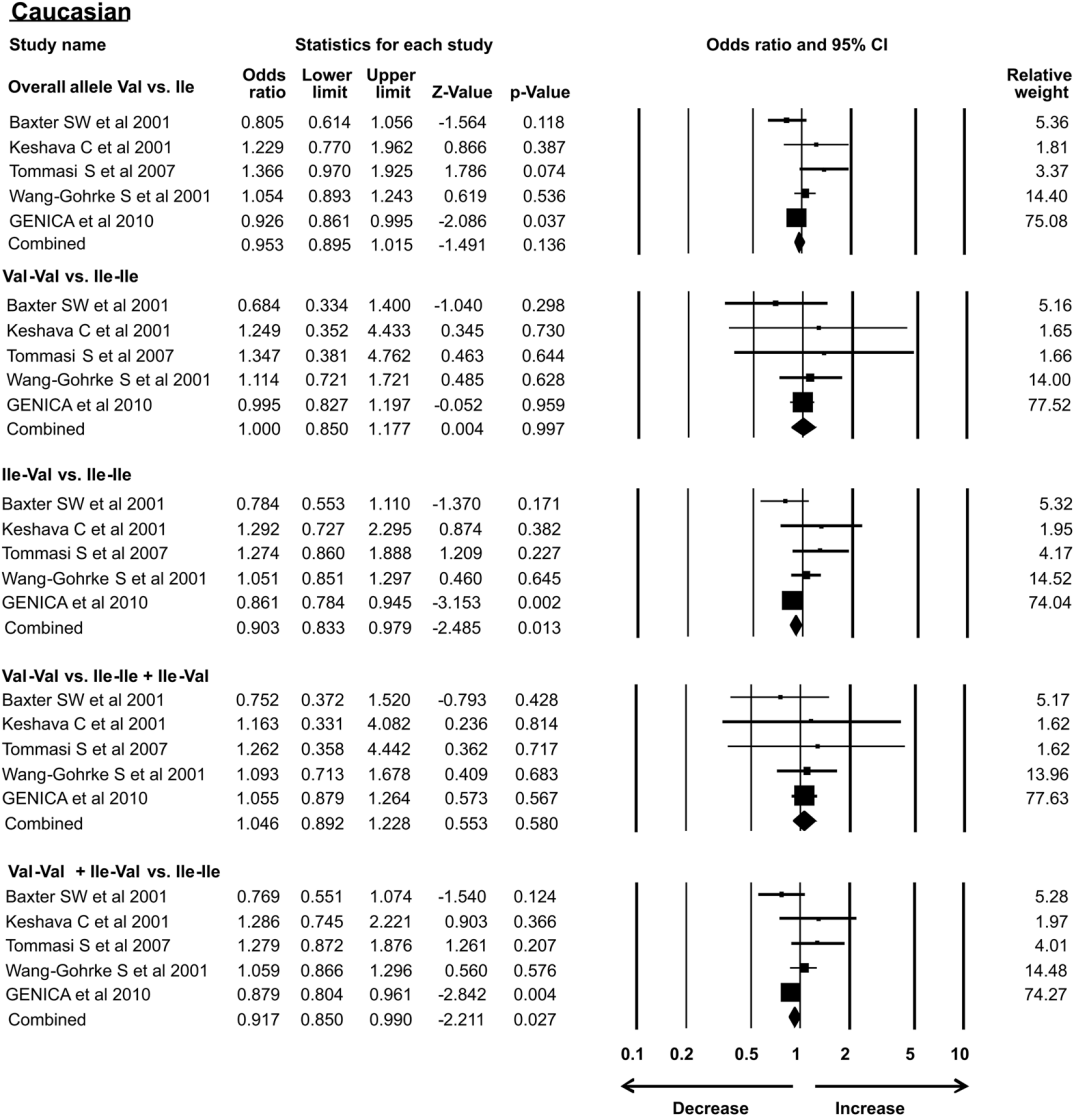
Forest plot: Analysis of *Her2* ^Ile^655^Val^ (rs1136201) gene polymorphism data from Caucasian ethnic group and validation of its correlation with breast cancer susceptibility using OR with 95% CI. Black squares represent the value of OR and horizontal line indicates 95% Confidence Interval (CI) of odds ratio (OR).

**Figure 8:**
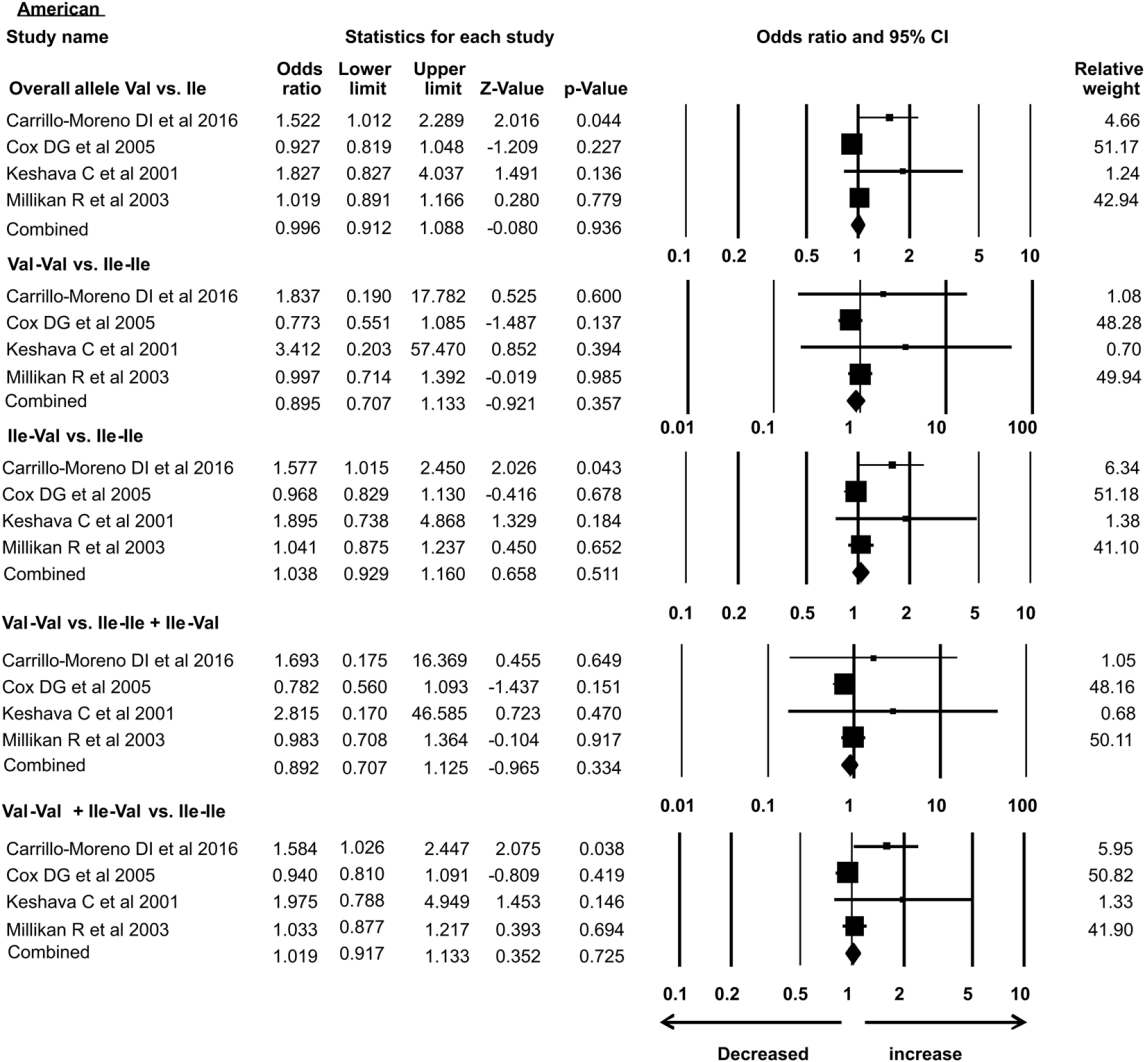
Forest plot: *Her2* ^Ile^655^Val^ (rs1136201) gene polymorphism data from American ethnic group showing OR and 95% CI for analyzing its association with breast cancer risk. Squares represents OR and horizontal line represents 95% Confidence Interval (CI) of odds ratio (OR).

**Figure 9:**
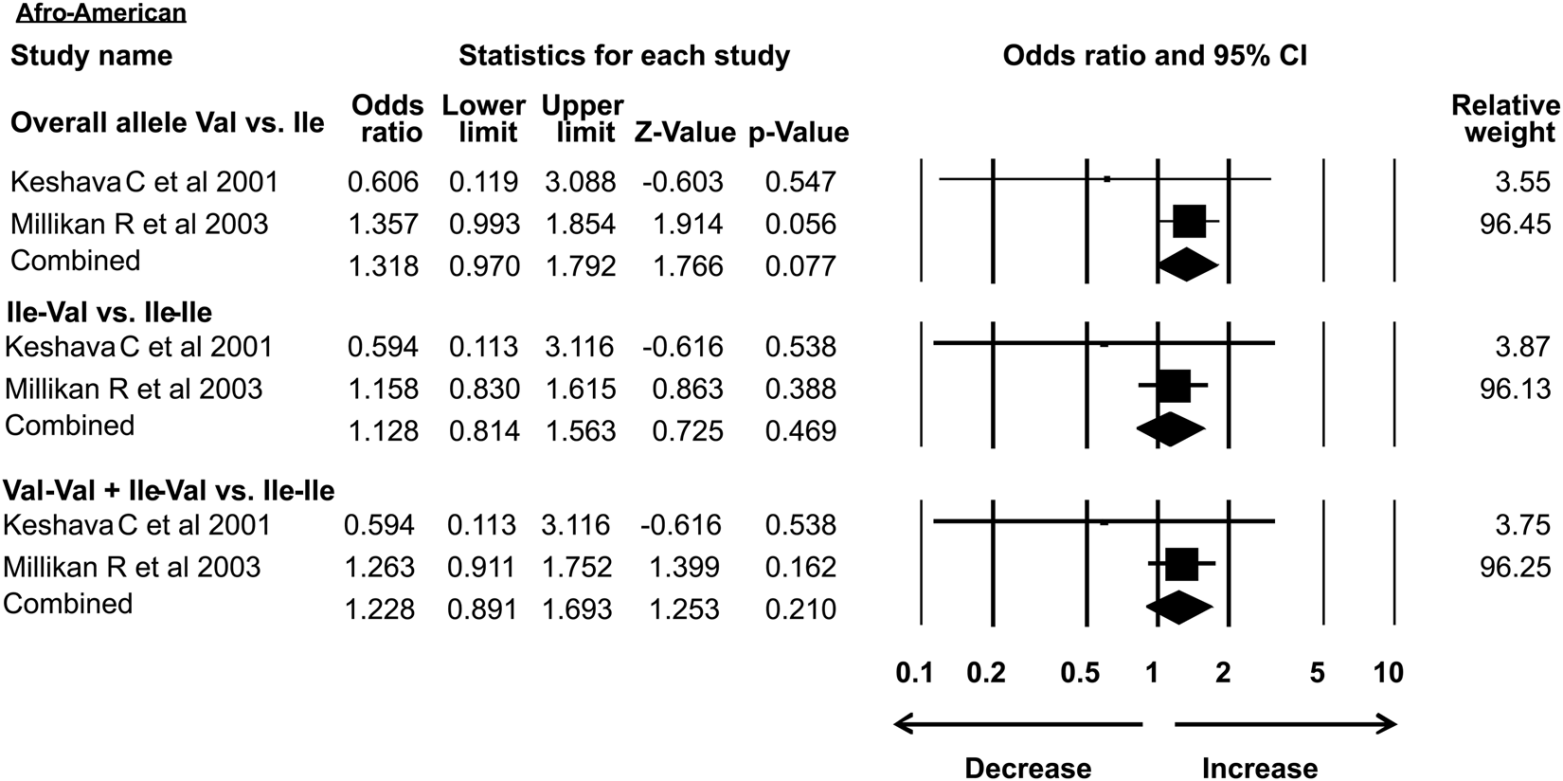
Forest plot: *Her2* ^Ile^655^Val^ (rs1136201) gene polymorphism data from Afro-American sub group population showing OR and 95% CI for validating its association with breast cancer risk. Black square represents OR and horizontal line representing 95% CI.

**Figure 10:**
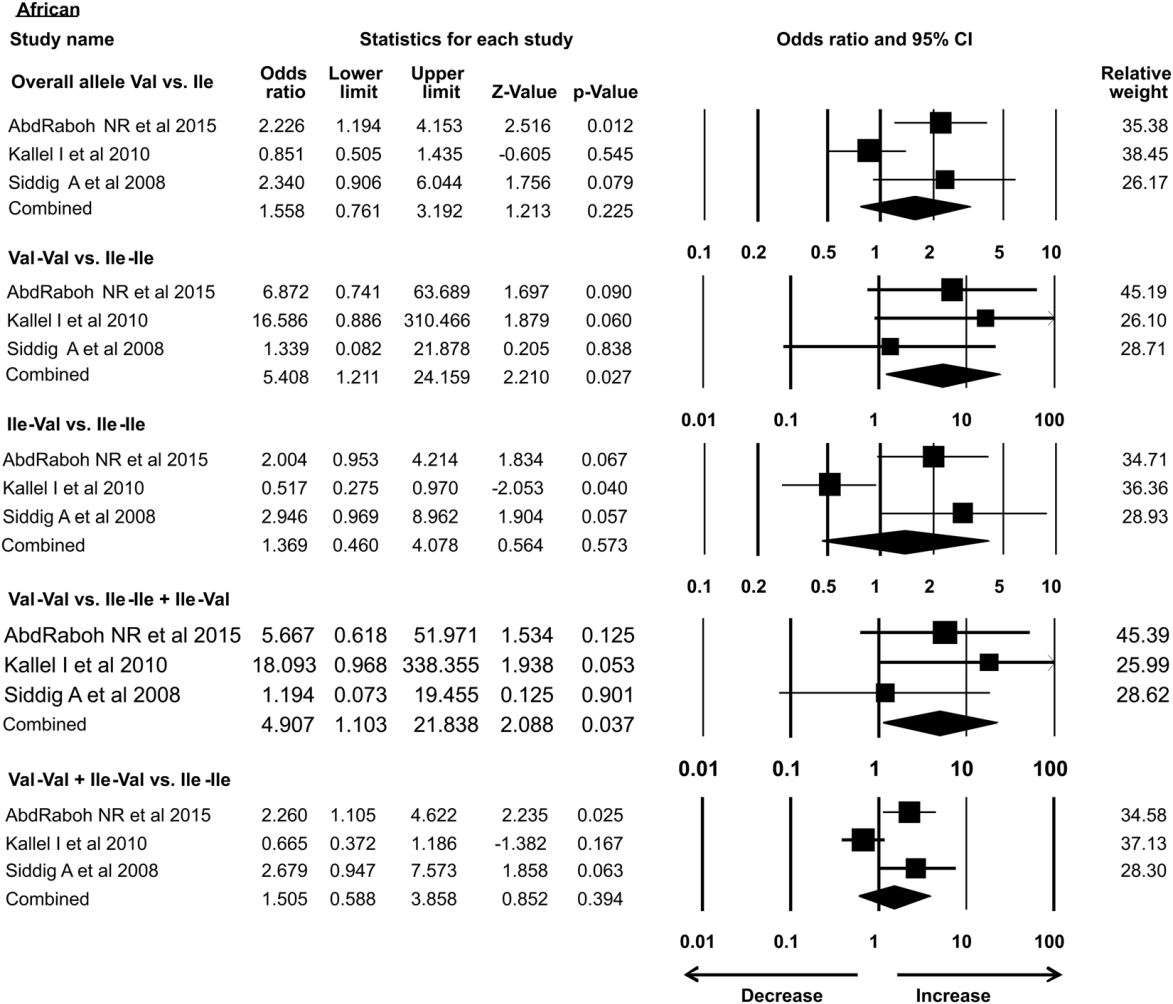
Forest plot: Overall analysis of African ethnic group *Her2* ^Ile^655^Val^ (rs1136201) gene polymorphism data for evaluation of its association with breast cancer susceptibility. Black squares represent the value of OR and horizontal line indicates 95% Confidence Interval (CI) of odds ratio (OR).

**Figure 11:**
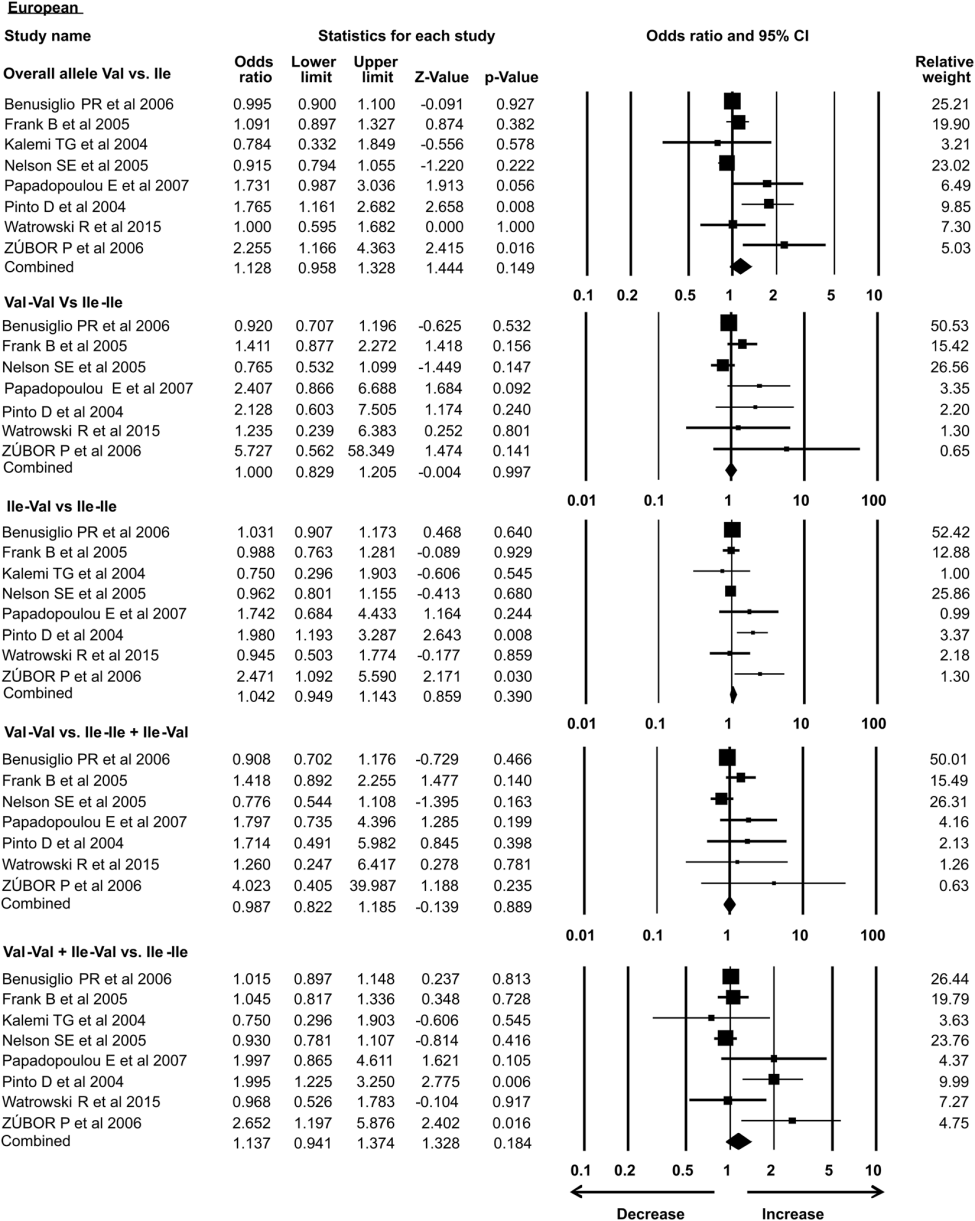
Forest plot: Overall analysis of *Her2* ^Ile^655^Val^ (rs1136201) gene polymorphism from European subgroup with OR and 95% CI for investigating the association with breast cancer risk. Black squares represent the value of OR and horizontal line indicates 95% Confidence Interval (CI) of odds ratio (OR).

**Figure 12:**
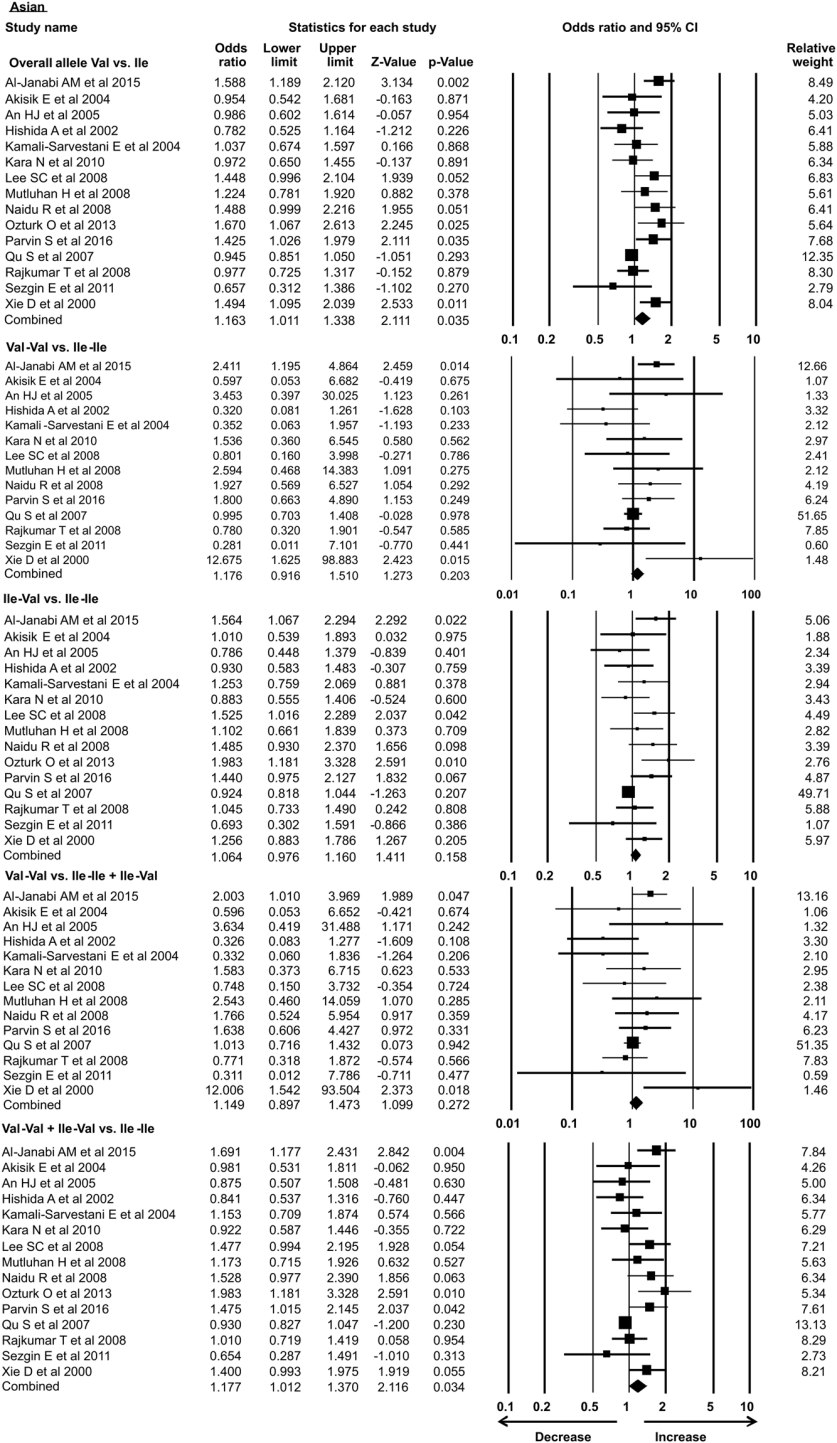
Forest plot: Overall analysis of *Her2* ^Ile^655^Val^ (rs1136201) gene polymorphism data from Asian ethnic group for the evaluation of association with breast cancer susceptibility. Black squares represent the value of OR and horizontal line indicates 95% Confidence Interval (CI) of odds ratio (OR).

As the previous meta-analysis presented association of *Her2* gene polymorphism with susceptibility to breast cancer in Asian population only, in the present analysis we re-accessed possible link of *Her2* polymorphism with BC in different ethnic groups. In our study subgroup analysis with 15 case-control studies identified the association with increased risk of breast cancer in Asian ethnicity in overall allele and dominant models. Similarly, African group with 3 successful included case-control studies also showed association with breast cancer risk in recessive and homozygous models. However, 5 case control studies from Caucasian, 4 from American subgroup, 2 limited studies from Afro-American ethnic group and 8 studies from European ethnicity showed no association of *Her2* polymorphism with breast cancer risk in all the models (Table 3) (Figs 7–12).

**Table 3:** Subgroup analysis of Her2 Ile 655 Val polymorphism and its association with breast cancer risk.

S.no	Model	Odds Ratio(OR)	95% CI	p-value
1	**Caucasian**	0.953	0.895–1.015	0.136
	Overall allele Val vs. Ile			
2	Homozygous Val-Val vs. Ile-Ile	1.000	0.850–1.177	0.997
3	Heterozygous Ile-Val vs. Ile-Ile	0.903	0.833–0.979	0.013
4	Recessive Val-Val vs. Ile-Ile + Ile-Val	1.046	0.892–1.228	0.580
5	Dominant Val-Val + Ile-Val vs. Ile-Ile	0.917	0.850–0.990	0.027
6	**American**	0.996	0.912–1.088	0.936
	Overall allele Val vs. Ile			
7	HomozygousVal-Val vs. Ile-Ile	0.895	0.707–1.133	0.357
8	Heterozygous Ile-Val vs. Ile-Ile	1.038	0.929–1.160	0.511
9	Recessive Val-Val vs. Ile-Ile + Ile-Val	0.892	0.707–1.125	0.334
10	Dominant Val-Val + Ile-Val vs. Ile-Ile	1.019	0.917–1.133	0.725
11	**Afro-American**	1.318	0.970–1.792	0.077
	Overall allele Val vs. Ile			
12	Homozygous Ile-Val vs. Ile-Ile	1.128	0.814–1.563	0.469
13	Dominant Val-Val + Ile-Val vs. Ile-Ile	1.228	0.891–1.693	0.210
13	**African**	1.558	0.761–3.192	0.225
	Overall allele Val vs. Ile			
14	Homozygous Val-Val vs. Ile-Ile	**5.408**	**1.211–24.159**	0.027*
15	Heterozygous Ile-Val vs. Ile-Ile	1.369	0.460–4.078	0.573
16	Recessive Val-Val vs. Ile-Ile + Ile-Val	**4.907**	**1.103–21.839**	0.037*
17	Dominant Val-Val + Ile-Val vs. Ile-Ile	1.505	0.588–3.858	0.394
18	**European**	1.128	0.958–1.328	0.149
	Overall allele Val vs. Ile			
19	Homozygous Val-Val vs. Ile-Ile	1.000	0.829–1.205	0.997
20	Heterozygous Ile-Val vs. Ile-Ile	1.042	0.949–1.143	0.390
21	Recessive Val-Val vs. Ile-Ile + Ile-Val	0.987	0.822–1.185	0.889
22	Dominant Val-Val + Ile-Val vs. Ile-Ile	1.137	0.941–1.374	0.184
23	**Asian**	**1.163**	**1.011–1.338**	0.035*
	Overall allele Val vs. Ile			
24	Homozygous Val-Val vs. Ile-Ile	1.176	0.916–1.510	0.203
25	Heterozygous Ile-Val vs. Ile-Ile	1.064	0.976–1.160	0.158
26	Recessive Val-Val vs. Ile-Ile + Ile-Val	1.149	0.897–1.473	0.272
27	Dominant Val-Val + Ile-Val vs. Ile-Ile	**1.177**	**1.012–1.370**	0.034*

### Sensitivity analysis

**Figure 13:**
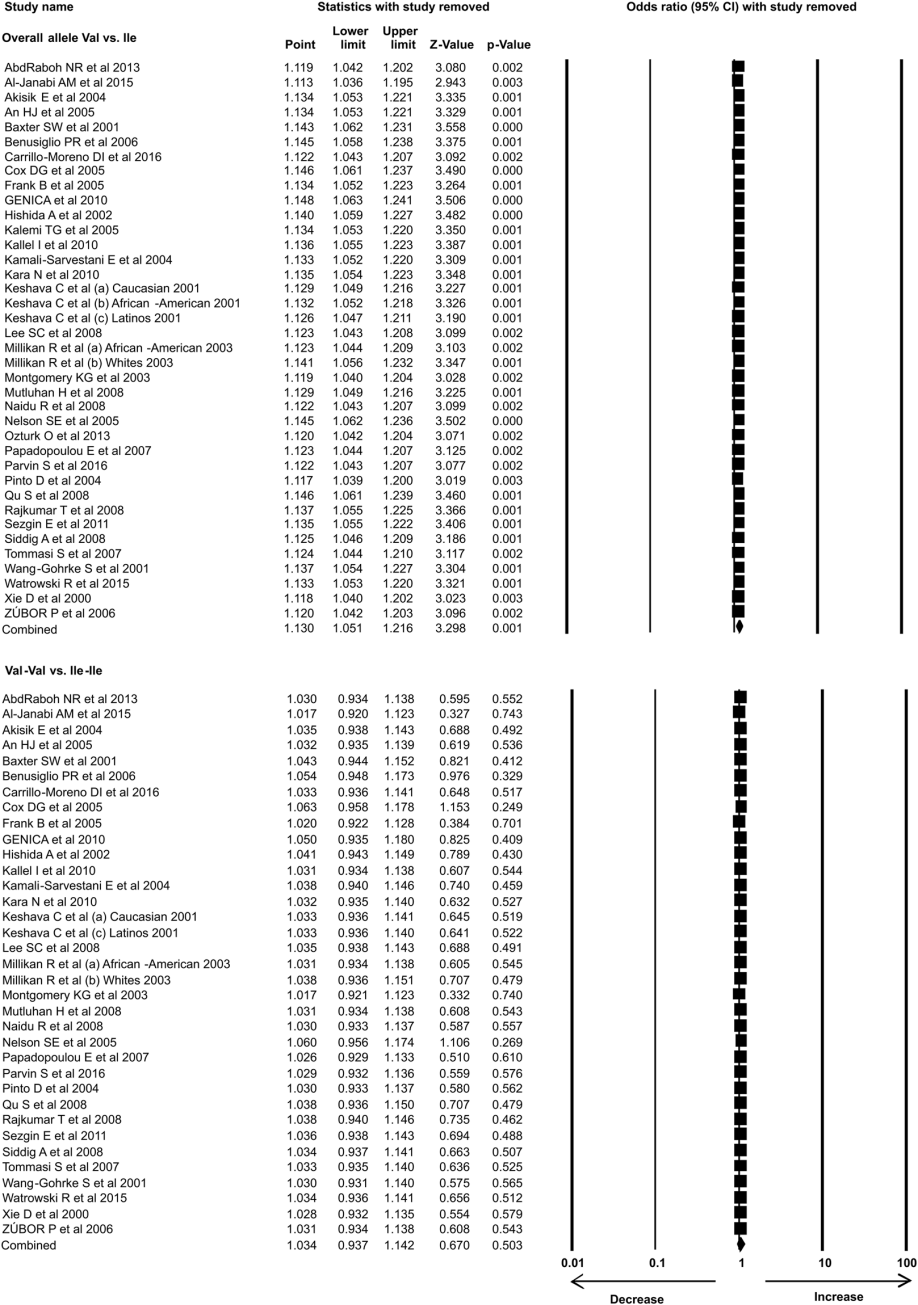
Sensitivity analysis: Sensitivity analysis showing no effect of single study on odds ratio (OR) in overall allele and genotypic (Homozygous: Val-Val vs Ile-Ile) analysis models.

**Figure 14:**
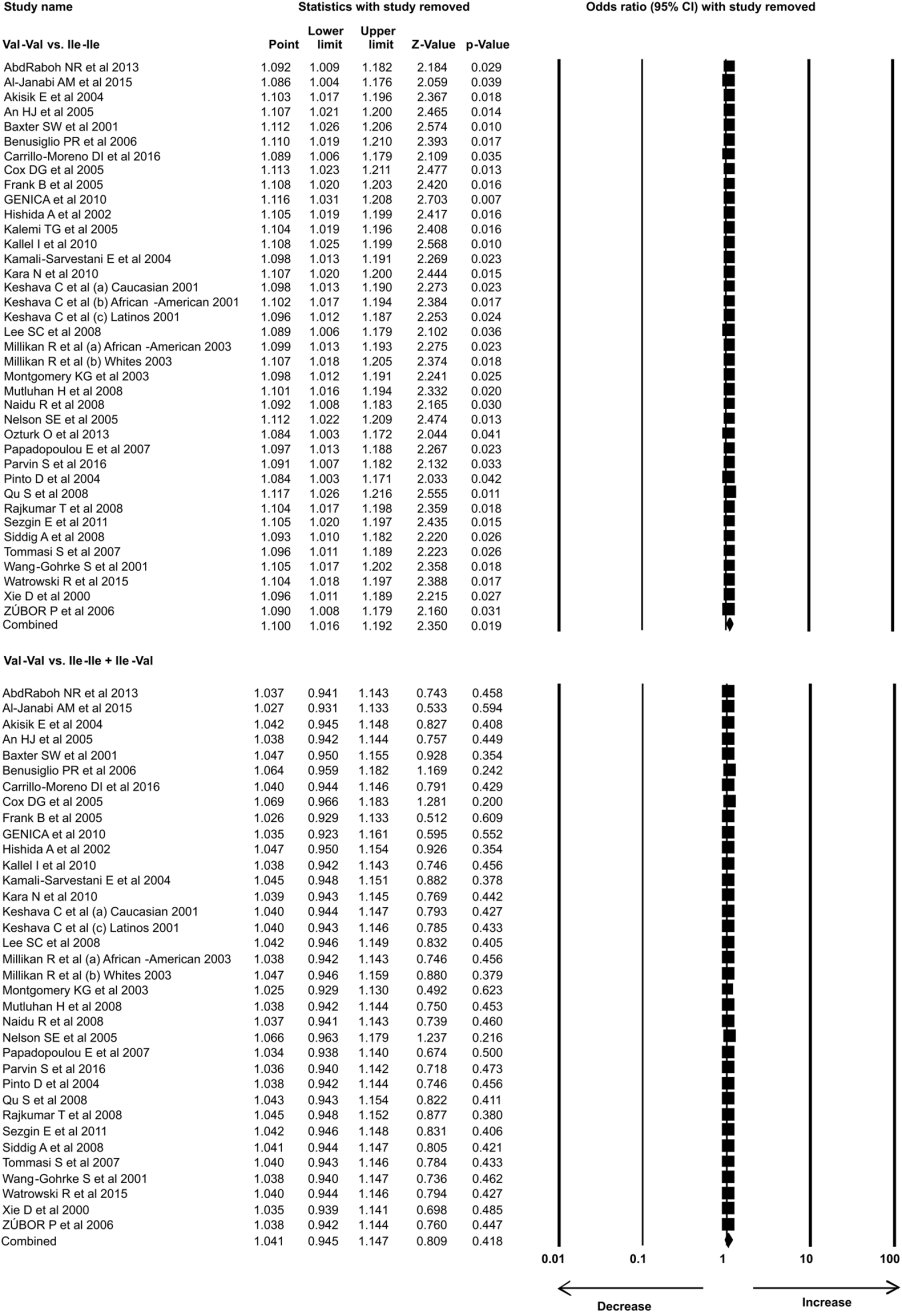
Sensitivity analysis: Sensitivity analysis with each study removal showing no effect on odds ratio (OR) in genotypic (Heterozygous: Ile-Val vs Ile-Ile and Recessive: Val-Val vs Ile-Ile + Ile-Val) analysis models of *Her2* ^Ile^655^Val^ (rs1136201) gene polymorphism.

**Figure 15:**
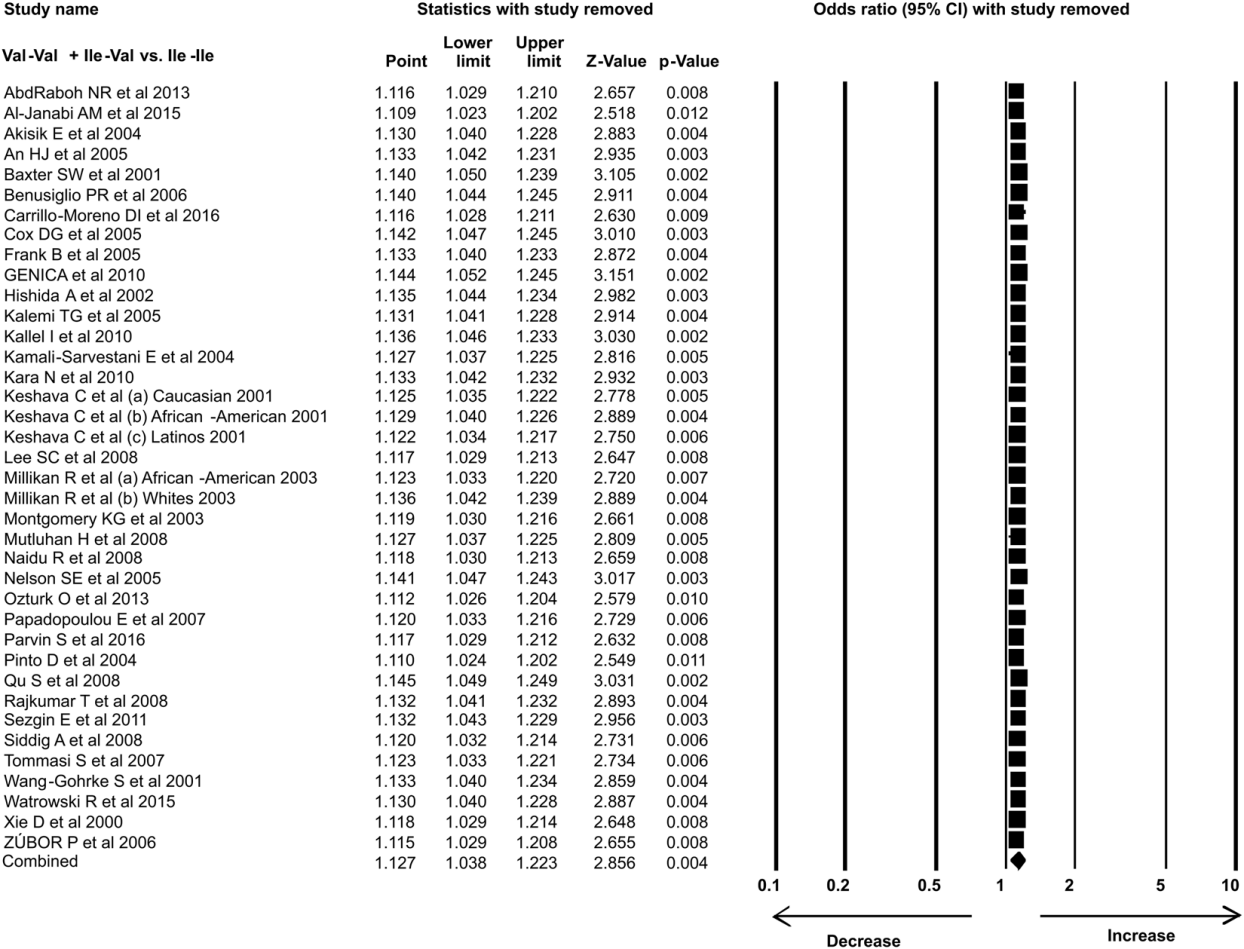
Sensitivity analysis: Sensitivity analysis showing no effect of single study on OR of genotypic (Dominant: Val-Val + Ile-Val vs Ile-Ile) analysis model of *Her2* ^Ile^655^Val^ (rs1136201) gene polymorphism.

We analyzed the influence of each individual study on the pooled OR by sensitivity analysis. One study was excluded each time and meta-analysis was performed. The results showed no individual study affected the pooled OR significantly, suggesting this meta-analysis is relatively credible, stable and not dependent on any individual study (Figs 13–15).

## Discussion

Human epidermal growth factor family members are a group of receptors with tyrosine kinase activity which affects cell proliferation and survival^63,64^. Dimerization of Her family members leads to autophosphorylation of tyrosine residues in the cytoplasmic domain and leads to cell proliferation and tumorigenesis^5–7^. Although Her family members lack natural ligand for signaling, various synthetic ligands have been developed and they are demonstrated to be efficientive in terms of drug delivery. Among the all-family members, Her2 is an important molecule and expression of *Her2* is elevated in various cancers^8–10^. Approximately 20–30% breast cancers show 40–100 fold elevated levels of *Her2*, whereas other cancer types such as ovarian, endometrial, gastric and esophageal cancers were also detected with over-expressed Her2 protein levels^65–71^. Single nucleotide polymorphisms (SNPs) are playing an important role in various cancer types and are capable of serving as diagnostic tools^23–25^. One such single nucleotide polymorphism with substitution of isoleucine with valine at codon 655 in transmembrane region of Her2 has been found to be playing an important role in development of cancer^72^. The transmembrane domain region of *Her2* with valine at 655 domain region stabilizes the formation of protein dimer and thus predisposing to an auto-activity of the receptor^73^. The hydrophobicity and conformational stability of the hydrophobic domains such as transmembrane domains may alter due to Isoleucine to valine change^74^. *Her2* ^Ile^655^Val^ polymorphism was well studied for association with breast cancer risk, whereas other polymorphism at 1170 codon of *Her2* (^Pro^1170^Ala^) was correlated with cardiotoxicity^75^. *Her2* ^Ile^655^Val^ polymorphism is not only associated with breast cancer risk but also associated with other cancers such as ovarian and endometrial cancers^76,77^. However, these results are inconsistent and a stringent and powerful analysis is required to conclude the association with breast cancer. In the present study we have analyzed the association of *Her2* ^Ile^655^Val^ polymorphism with increased breast cancer susceptibility using powerful tool comprehensive meta-analysis (CMA). Overall allele comparison genetic model results suggest that valine allele in *Her2* 655 codon favors the development of breast cancer in worldwide population. Heterozygous, dominant models also prove that *Her2* polymorphism is associated with increased risk of breast cancer. Whereas, subgroup analysis showing different results for different ethnic population. Earlier reports by Wang *et al*.^58^ and Chen *et al*.^62^ demonstrated association of *Her2* valine allele with breast cancer risk in Caucasian population. In contrast, our study failed to show such link. The present study has several advantages over earlier reports. We have included more number of studies in the current meta-analysis including larger number of cases and controls.

Tao *et al*.^56^ reported the association of *Her2* polymorphism with breast cancer risk in Asian population whereas later Wang *et al*.^58^ and Chen *et al*.^62^ showed no such association with breast cancer. In this present meta-analysis, we have performed subgroup analysis and demonstrated that valine allele is associated with breast cancer risk in Asian population. In addition to that Val-Val + Ile-Val vs Ile-Ile model also prove the susceptibility of *Her2* polymorphism with breast cancer. We also observed that subjects with valine/valine genotype are susceptible for the development of breast cancer in African population. These results are in agreement with the study demonstrated by Wang *et al*.^58^; however other studies failed to show such association with breast cancer susceptibility. Ethnic groups such as American, European and Afro-American are not showing such association with breast cancer risk. Our present meta-analysis includes all the studies in which either Taqman or RFLP used as detection method. Frank *et al*. suggested the biasness in the methods used for the detection of polymorphism and suggested that Taqman method is capable of producing false results^78^. We excluded the studies in which Taqman method used as detection method and performed the analysis. Studies which used other than Taqman method for the detection of polymorphism showed significant association with breast cancer risk in all models.

In conclusion, our present meta-analysis demonstrated that valine allele is susceptible in overall worldwide population and Asian ethnic group. *Her2* ^Ile^655^Val ^polymorphism is associated with breast cancer risk in Asian, African population but not in other ethnic groups such as Caucasian, European, American and Afro-American. These results suggest that *Her2* ^Ile^655^Val^ polymorphism could be considered as possible susceptible bio marker for the detection of breast cancer.

## Materials and Methods

### Literature search and identification of relevant studies

A systematic extensive search was performed to extract the appropriate published reports using online databases i.e., Pubmed, EMBASE and Google scholar. The publication search was performed by three independent authors (BMK, SC& DRM) using either single or combination of given keywords i.e., “*Her2* ^Ile^655^Val^ polymorphism”, “Herceptin receptor polymorphism”, “rs1136201” and breast cancer. In addition to the preliminary online database search we have checked the cross references for the potential publications, those possibly missed in preliminary search. Our present study includes recently published (earliest by 2017) 35 case-control studies with 19, 220 cases and 22, 306 controls for *Her2* ^Ile^655^Val^ polymorphism (Supplementary Fig. 2).

### Inclusion and exclusion of studies

The studies which met all the criteria given below have been included in the present meta-analysis: (a) studies published in English, (b) must have case-control or cohort design, (c) have available genotype frequency of both the cases and controls or have odds ratio (OR) and 95% confidence interval (CI) values, (d) evaluating the association of *Her2* ^Ile^655^Val^ polymorphism with breast cancer risk and (e) studies representing original data. The studies excluded based on the criteria given below: (a) studies published in other languages except English, (b) studies having only case samples, (c) representing risk of other cancers, (d) without genotypic distribution and allele frequency data and (e) reviews and abstracts.

### Data extraction

The data extraction was performed by three independent authors (BMK, SC & DRM) independently and the disagreement about the studies between the authors was resolved and came to a conclusion by conducting a group discussion within the authors. We followed previously established data form to extract the data from the studies and the following data was extracted from each article: first author’s name, year of publication, country, ethnicity, number of case and control samples, genotype distribution, allele frequency for each case and control.

### Meta-analysis

The current meta-analysis was performed using comprehensive meta-analysis version 3 software (CMA v3) https://www.meta-analysis.com/pages/comparisons.php. CMA v3 is a powerful tool to analyze and has several advantages over other software available for computational meta-analysis. Combined odds ratio with 95% CI was calculated and was taken into consideration to apprise the association of *Her2* polymorphism with breast cancer risk. Chi-Squared based Q test was performed to analyze the heterogeneity and p-value < 0.05 was considered as significant. In case of no significant heterogeneity fixed effect model was used to assess the combined OR. In contrast, Random effect model was considered to calculate the combined odds ratio with 95% CI among the studies. I^2^ statistics was used to quantify inter study variability, greater I^2^ value depicts greater degree of heterogeneity. Publication bias was examined using Begg’s funnel plot. Egger’s linear regression test was employed to analyze and measure the asymmetry of Begg’s funnel plot and the significance of intercept was assessed by t-test. Intercept considering p-value < 0.05 was considered as significant and the publication bias was reduced using “trim and fill” method.

### Availability of data and materials

All those named as authors confirmed the availability of data and materials.

## Electronic supplementary material

Supplementary Info
