# Supplementary material for: Her2Ile655Val polymorphism and its association with breast cancer risk: an updated meta-analysis of case-control studies
Source: Sci Rep. 2018 May 9;8:7427. doi: 10.1038/s41598-018-25769-y (PMC5943262; doi:10.1038/s41598-018-25769-y)
Supplement: Supplementary file 1 — Supplementary Info [file 41598_2018_25769_MOESM1_ESM.pdf]

Title: - *Her2* $^{Ile}655^{Val}$ polymorphism and its association with breast cancer risk: an updated meta-analysis of case-control studies.

B Madhu Krishna[1], Sanjib Chaudhary[1], Aditya K Panda[2], Dipti Ranjan Mishra[3], Sandip K Mishra[1*]

1. Cancer Biology Lab, Gene Function & Regulation Group, Institute of Life Sciences, Nalco square, Chandrasekharpur, Bhubaneswar, 751023, Odisha, India.

2. Centre for Life Sciences, Central University of Jharkhand, Ranchi, 835205, Jharkhand, India.

3. Gene Function & Regulation Group, Institute of Life Sciences, Nalco square, Chandrasekharpur, Bhubaneswar, 751023, Odisha, India.

Supplementary Figure 1

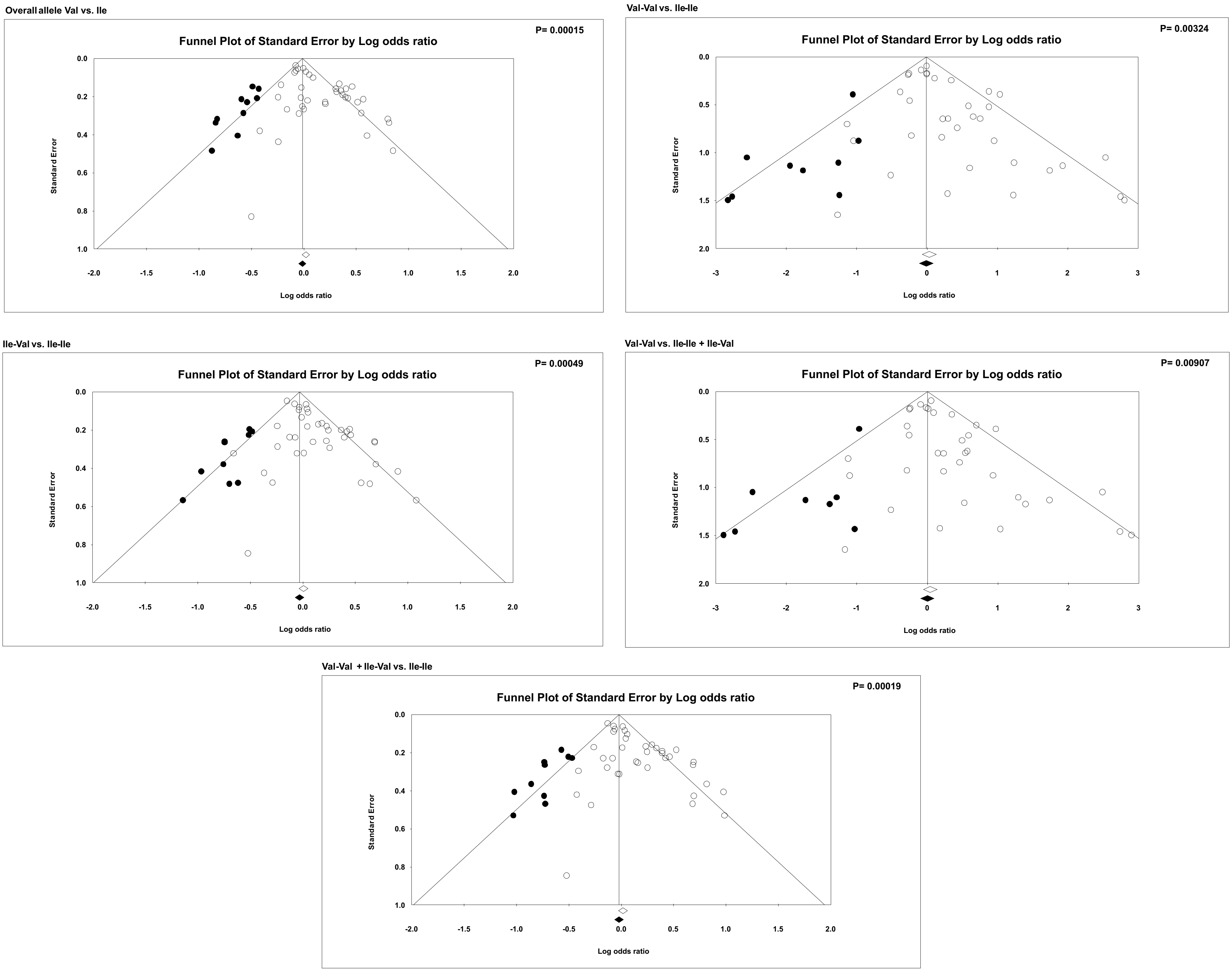

Supplementary Figure 1. Funnel Plot: Publication biasness was analyzed in each and every model, “Trim and fill” method was used to remove publication bias.

Supplementary Figure 2

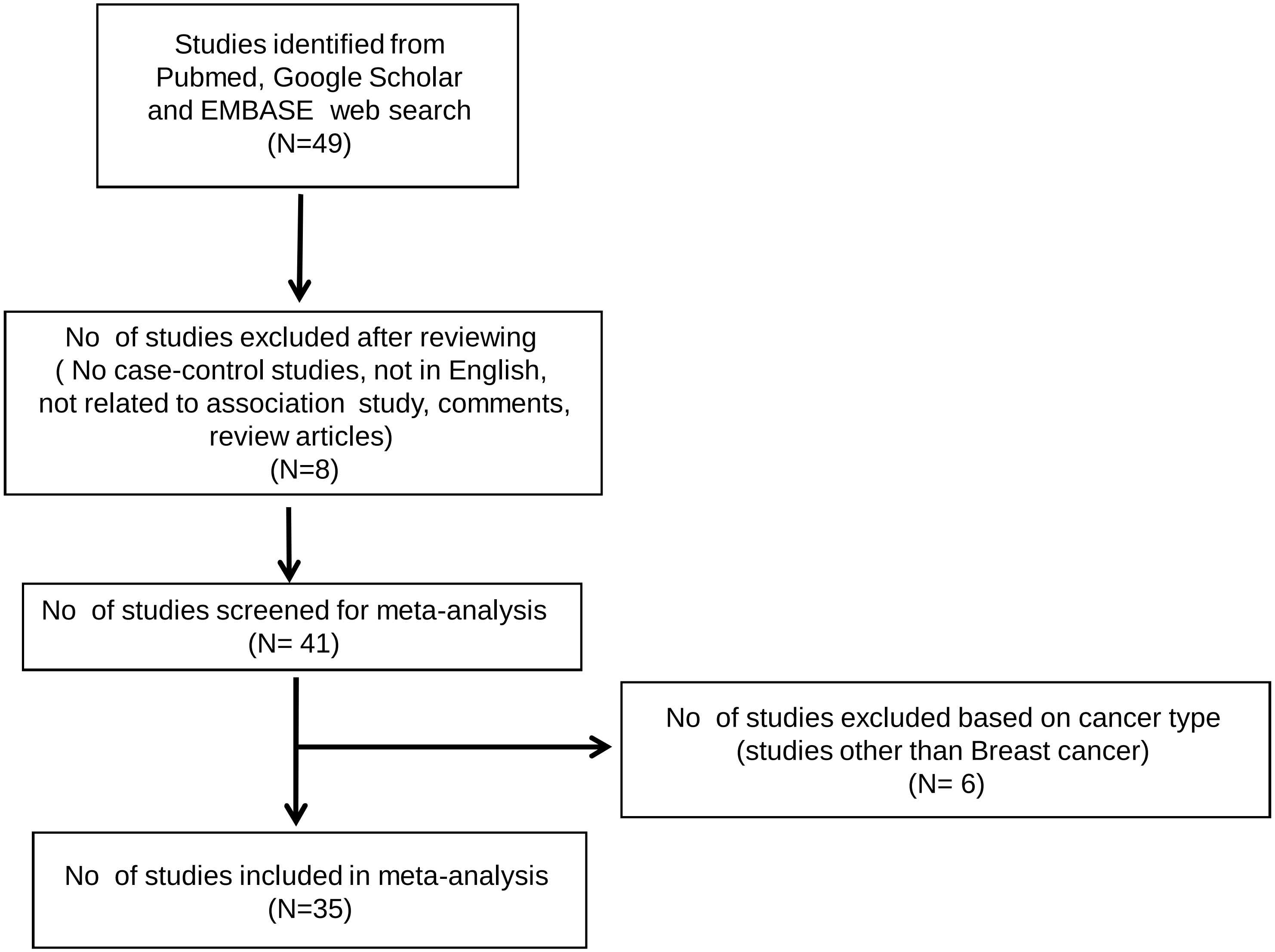

Supplementary Figure 2. Prisma chart: Prisma chart showing the exclusion and inclusion of studies used for meta-analysis.
